# Mechanics of Reversible Deformation during Leaf Movement and Regulation of Pulvinus Development in Legumes

**DOI:** 10.3390/ijms231810240

**Published:** 2022-09-06

**Authors:** Miyuki T. Nakata, Masahiro Takahara

**Affiliations:** 1 Nara Institute of Science and Technology, Ikoma 630-0192, Nara, Japan; 2 Acacia Horticulture, Kizugawa 619-0224, Kyoto, Japan

**Keywords:** adaxial/abaxial identity, bending moment, cellulose microfibrils, cell wall, ELP1/PLP, leaf movement, motor cells, pulvinus, reversible deformation, turgor pressure

## Abstract

Plant cell deformation is a mechanical process that is driven by differences in the osmotic pressure inside and outside of the cell and is influenced by cell wall properties. Legume leaf movements result from reversible deformation of pulvinar motor cells. Reversible cell deformation is an elastic process distinct from the irreversible cell growth of developing organs. Here, we begin with a review of the basic mathematics of cell volume changes, cell wall function, and the mechanics of bending deformation at a macro scale. Next, we summarize the findings of recent molecular genetic studies of pulvinar development. We then review the mechanisms of the adaxial/abaxial patterning because pulvinar bending deformation depends on the differences in mechanical properties and physiological responses of motor cells on the adaxial versus abaxial sides of the pulvinus. Intriguingly, pulvini simultaneously encompass morphological symmetry and functional asymmetry along the adaxial/abaxial axis. This review provides an introduction to leaf movement and reversible deformation from the perspective of mechanics and molecular genetics.

## 1. Introduction

**Figure 1:**
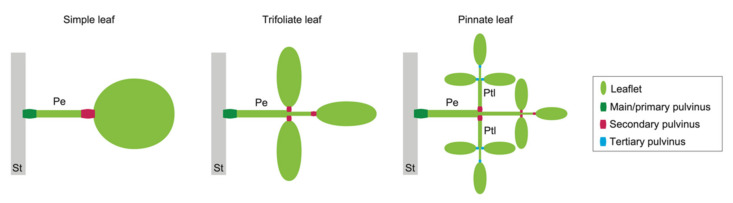
Examples of pulvini in three types of leaves. A simple leaf (**left**) has pulvini at the two ends of a petiole. A trifoliate leaf (**middle**) has pulvini at the base of a petiole and of leaflets. A pinnate leaf (**right**) has pulvini at the base of petiolules as well as at that of a petiole and of leaflets. St, a stem; Pe, a petiole; Ptl, a petiolule.

The repeated and reversible deformation of organs is a survival strategy seen in a variety of plants. Reversible cell deformation in plants differs in mechanical principle from irreversible cell growth. In other words, irreversible cell growth progresses continuously by stress relaxation caused by cell wall creep, whereas reversible cell deformation is based on the elastic properties of the cell wall that are sensitive to changes in turgor pressure. A prominent example of repeated and reversible deformation is the leaf movements of legumes. The leaf movement of legume species is actuated by a repetitive bending deformation of a pulvinus, a joint-like thickening at the base of a petiole, of a petiolule, or of a leaflet (Figure 1). The deformation of a pulvinus is driven by parenchyma cells, called “motor cells.” To lower the leaf, the motor cells on the upper side of the pulvinus expand and those on the lower side contract in a coordinated manner; the opposite occurs to raise the leaf.

Understanding the mechanisms of dynamic leaf movement and pulvinar deformation requires a basic knowledge of an interdisciplinary field that includes physics, chemistry, and biology. As an introduction for biologists who are not experts in leaf movement, this review begins by outlining the basic mathematics that describe changes in cell volume, the function of the cell wall, and the mechanics of bending deformation. Next, we summarize recent molecular genetic studies of pulvinar identity and function. Finally, we review the adaxial–abaxial patterning in early leaf development that determines the cell properties on the upper (adaxial) and lower (abaxial) sides of the leaf and discuss the potential role of this patterning in motor cell function. This review presents unsolved mysteries of leaf movement and pulvinus function, especially from the perspectives of mechanics, development, and evolution.

## 2. Mathematics of Changes in Cell Volume

Changes in cell volume are triggered by the net flow of water into or out of the cell. Living cells have multiple compartments surrounded by biological membranes, such as cell and organelle membranes. Biological membranes consist of a lipid bilayer in which functional proteins, including channel proteins, are embedded. Channel proteins control the movement (import and export) of substances between the inside and outside of the membrane. Therefore, a biological membrane can function like a semipermeable membrane and can form a concentration gradient of solutes (K^+^, Cl^−^, H^+^, etc.) between the inside and outside of a compartment. Solute concentration gradients drive the inflow or outflow of water via the water channel aquaporin. Since, in plant cells, cell size often correlates with the size of the vacuole, the characteristics of the plasma membrane and the vacuolar membrane (tonoplast) both are important variables influencing changes in cell volume, but here, for convenience, we consider only the plasma membrane. The rate of water flow between the inside and the outside of the cell, *J_v_*, is defined by the product of the hydraulic conductivity, *L_p_*, which is related to the function of aquaporin, and the difference in water potential Ψ between the inside and outside of the cell [1,2].

(1) $$J_{v}=L_{p}\cdot ∆\Psi$$

(2) $$∆\Psi =\Psi_{outer}-\Psi_{inner}$$

where Ψ*_outer_* and Ψ*_inner_* are the extracellular and intracellular water potentials, respectively.

**Figure 2:**
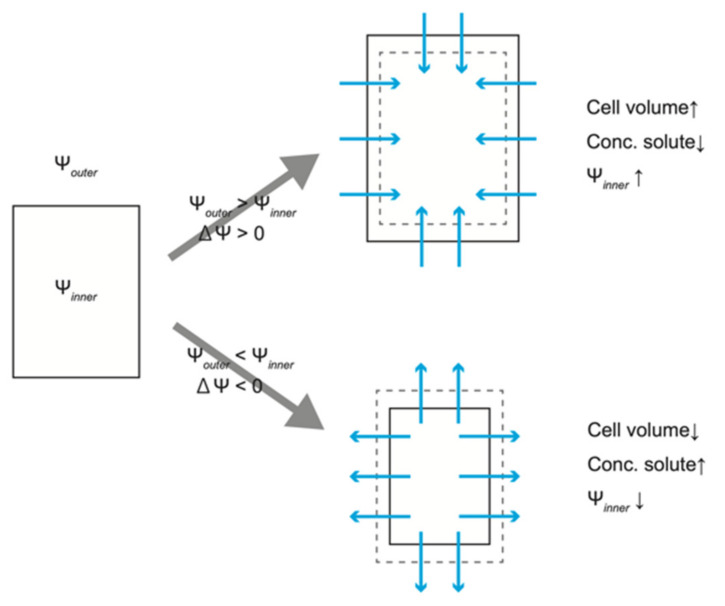
Schematic view of the relationship between water potential and cell volume.

Thus, the rate of water flow between the inside and the outside of the cell is proportional to the difference between the extracellular and intracellular water potentials. When there is a difference in water potential due to a difference in solute concentration inside and outside of the cell, water flows from the side with the higher water potential to the side with the lower water potential (Figure 2). An influx of water causes an increase in cell volume, lowering the solute concentration inside the cell, and resulting in an increase in water potential. On the other hand, an outflow of water reduces cell volume and increases the solute concentration in the cell, resulting in a decrease in water potential. Water flow continues until the intracellular water potential reaches the same value as that of the extracellular water potential (i.e., ∆Ψ = 0). Since plant cells have a cell wall, the water potential gradient resulting from significant differences in solute concentration is counteracted by the pressure potential caused by the tensile force of the cell wall. This is called “turgor pressure” in the field of plant science. The potential caused by the difference in solute concentration is distinguished from turgor pressure as “osmotic potential”, and the intracellular water potential Ψ*_inner_* is expressed as the sum of the osmotic potential Ψ*_π_* and the turgor pressure *P* [1,2].

(3) $$\Psi_{inner}=\Psi_{\pi }+P$$

More generally, the water potential also includes two other components: the movement of water in the direction of gravity (i.e., gravitational potential) and that between plant cells and solid particles in the soil (i.e., matrix potential). The effect of these potentials is, however, negligible when considering the microscopic movements of water in internal tissues. In summary, in the case of water flow into plant cells, when the intracellular water potential—that is, the sum of the osmotic potential and turgor pressure—reaches the same value as the extracellular water potential (= osmotic potential), the net flow of water appears to stop (*J_v_* = 0 because ∆Ψ = 0), resulting in an equilibrium state.

Cellulose microfibrils are the main source of cell wall tensile strength. Bundled cellulose microfibrils can exert a strong tensile force. The average angle (orientation) of cellulose microfibrils can be biased in a particular direction in cells of some tissues. This bias causes anisotropic extension when the cell volume increases. Cellulose microfibrils are cross-linked by hemicellulose to form a network, and the gaps are filled with matrix components such as pectin to maintain integrity. Cellulose microfibrils exhibit a very high tensile rigidity in the direction parallel to the fiber. In a previous report, a synchrotron X-ray diffraction analysis was used to estimate changes in the angle of cellulose microfibrils during a tensile test of compression wood, demonstrating that tension had a negative proportional relationship with the angle and strain of cellulose microfibrils [3]. Therefore, cellulose microfibrils appear to behave like springs during cell elongation. In pulvinar motor cells, the orientation of cellulose microfibrils is almost perpendicular to the proximodistal axis [4,5], which is consistent with the extension anisotropy of pulvinar tissue slices and cells, namely, their tendency to elongate parallel to the proximodistal axis [4,5,6]. In addition, the overexpression of *Populus alba* derived cellulase in *Paraserianthes falcataria* caused a delayed initiation of leaf closure [7], demonstrating the importance of cellulose in the regulation of leaf movement. The deformation of an object includes a reversible elastic deformation, which is caused by forces weaker than the yield point of the object, and a permanent plastic deformation, which is caused by forces exceeding the yield point. In both elastic and plastic deformations, cellulose microfibrils are stretched like springs but do not fracture because of their strength. According to studies of compression wood, it has been suggested that shearing occurs between cellulose microfibrils during plastic deformation [3], while the matrix structure is maintained during elastic deformation. If repeated extension and contraction involved plastic deformation, it would be necessary to assume a physiological mechanism that “remembers” the original state of the matrix and restores it after shearing. Therefore, the reversible deformation of plant organs is traditionally modeled as an elastic deformation of the cell wall by forces that do not exceed the yield point.

The equations describing the relationship between the elastic properties of the plant cell wall and cell volume changes are as follows [1]:

(4) $$\varepsilon =\frac{dP}{dV}\cdot V=\frac{dP/dt}{dV/dt}\cdot V$$

(5) $$\frac{1}{V}\frac{dV}{dt}=\frac{1}{\varepsilon }\frac{dP}{dt}$$

where ε corresponds to the volumetric elastic modulus or bulk modulus of the cell wall, which indicates the difficulty of volume change when a pressure is applied. *V* corresponds to the cell volume, *dV/dt* corresponds to the change in cell volume per unit time, and *dP/dt* corresponds to the change in turgor pressure per unit time. The left side of Equation (5) shows the relative change in cell volume per unit time. This equation indicates that the relative change in cell volume depends on both the change in turgor pressure and the mechanical properties of the cell wall. The bulk modulus of the cell wall is a combination of factors such as cell wall thickness; Young’s modulus, which represents the resistance to deformation of the material; and Poisson’s ratio, which represents the ratio of the strain in the same direction as the loading and the deformation perpendicular to the direction of the loading when loading to one direction [1]. Using this equation, the bulk modulus can be estimated by measuring the change in volume and turgor pressure after a change in the extracellular water potential. The bulk modulus of the *Phaseolus coccineus* pulvinus has been estimated to be approximately 1 MPa based on the results of immersing tissue slices containing pulvinar motor cells in solutions of various mannitol concentrations [4].

Cell volume changes by the combination of elastic and plastic deformations are represented by the Lockhart–Ortega equation [8],

(6) $$\frac{1}{V}\frac{dV}{dt}=\frac{1}{\varepsilon }\frac{dP}{dt}+\Phi \left[P-P_{c}\right]$$

where *P_c_* corresponds to the turgor pressure at the yield point, and Φ is a constant representing the extensibility of the cell wall [8]. $\Phi \left[P-P_{c}\right]$ is positive if *P* exceeds *P_c_*, and zero otherwise. Note that the first term on the right side of Equation (6) is the same as that on the right side of Equation (5). When *P_c_* is sufficiently large, cell volume changes in vivo require only an elastic deformation, so that theoretically, a reversible deformation can be repeated. That is, assuming that the reversible deformation involves only an elastic deformation, the conditions that enable such deformation are that (1) the value of ε is sufficiently small and/or (2) the value of *P_c_* is sufficiently large. The orientation of cellulose microfibrils is the most important determinant of ε; in line with this, as described above, the cellulose microfibrils of pulvinar motor cells are oriented perpendicular to the elongation direction, minimizing the ε of the elongation direction. ε and *P_c_* can also be affected by the matrix structure between the cellulose microfibrils. Our recent study [9] found that the pulvinus was rich in pectin compared to other axial organs. The cell walls of pulvinar motor cells also had many slits (pulvinar slits) running perpendicular to the proximodistal axis, in which the amount of cellulose was low and de-methyl-esterified pectin was abundant. Computer simulations indicated that the slits were capable of converting changes in turgor pressure into cellular deformation along an axis perpendicular to the slits [9]. The association between pulvinar slits and the proximodistal-biased deformation of pulvinar motor cells is further supported by the results of a mechanical test and the finding that pulvinar slits are widely conserved in legumes [9]. Meanwhile, a mechanical test of the onion epidermis after enzymatic pectin esterification indicated that the de-methyl-esterification of pectin increased the plasticity but not the elasticity of the cell wall [10]. What are the relative contributions of the elastic deformation and plastic deformation to the reversible deformation of motor cells? What roles do pectin and pulvinar slits play in the reversible deformation of pulvinar motor cells? These are intriguing questions for future research into the mechanics of reversible cell deformation.

## 3. Mechanics of Bending Deformation

To connect microscopic changes in cell volume to macroscopic organ deformation, the volume changes must be synchronized within a cell population. In the case of leaf movement, a synchronized change in volume of the upper motor cells is accompanied by the opposite change in the lower ones. Bending deformation caused by changes in the turgor pressure of motor cells appears to be morphologically similar to that caused by an external force. This section begins with an outline of the mechanics of bending deformation and then explains how pulvinar deformation is described in equations.

**Figure 3:**
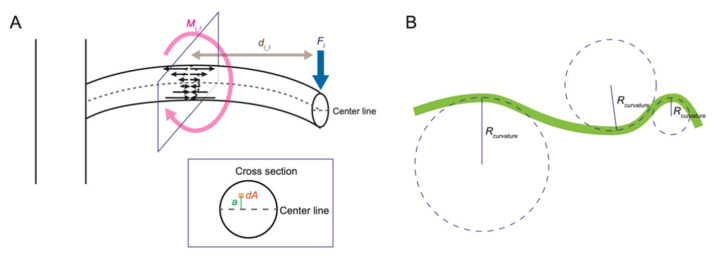
Mechanical models of the bending deformation. (**A**) A cantilever model of bending deformation. Arrows indicate the degree of expansion or contraction inside an object. (**B**) Schematic views of the degree of bending modeled as an arc of virtual circles of varying size.

The leaves and branches of a plant are mechanically unstable structures connected to the main stem only at their base and are often modeled as cantilever beams (Figure 3A). When an external force (load) is applied perpendicularly to the long axis of a cantilever, a bending force and a shear force are generated at each point on the cantilever. Here, we focus on the bending force. In this model, the bending force is described as the bending moment. When a load *F_l_* is applied to point *l* of a cantilever, the bending moment *M_i_l_* at a point *i* is expressed by the product of the distance *d_i_l_* between the points *i* and *l* and *F_l_*.

(7) $$M_{i\_l}=d_{i\_l}\cdot F_{l}$$

The relationship between the bending moment and the degree of bending can be described using a cantilever model in which the degree of bending is modeled as an arc of virtual circles of varying size (Figure 3B). A large circle shows a small degree of bending, while a small circle shows a large degree of bending. The radius of these virtual circles is called “the radius of curvature”, and the inverse of the radius of curvature is “the curvature”. When the same bending moment is applied to two rods with different stiffness, the degree of bending of the stiffer one is smaller than that of the more flexible one. The value that expresses the difficulty of bending deformation is the flexural rigidity *EI*. *EI* is the product of Young’s modulus *E* and the moment of inertia of area *I*, which reflects the effect of the two-dimensional arrangement of the material in the cross section. In the cantilever model, when bending deformation occurs, the area near the center line does not expand or contract, and the degree of expansion or contraction increases as the distance from the center line increases (Figure 3A). *I* represents the influence of the size and the shape of the cross section on bending and is defined as the integral value of the product of the square of the distance *a* from the center line and the derivative of the area *dA*.

(8) $$I=\int a^{2}dA$$

The value of *I* is formulated for idealized cross-sectional shapes such as circles and rectangles [11]. If an object is composed of *n* kinds of materials, the flexural rigidity *EI* of the object is the sum of the flexural rigidity of each material *E_j_I_j_*.

(9) $$EI=\sum_{j=1}^{n}E_{j}I_{j}$$

The relationship between the bending moment *M* and *EI* and the radius of curvature *R_curvature_* or curvature *κ* is expressed by the following equation:

(10) $$M=\frac{EI}{R_{curvature}}=EI\cdot \kappa$$

Pulvinar bending deformation is modeled using a deflection torque [12]. When the pulvinus is deformed, the deflection torque is estimated as the product of the measured value *F_i_*, the force that the petiole generates in the rotational direction at the point *i*, and the distance *d_i_p_* from the point *i* to the position of the pulvinus *p*. Therefore, from Equation (7), the deflection torque is equivalent to the bending moment *M*.

(11) $$deflection\;torque=F_{i}\cdot d_{i\_p}(=M)\;$$

In this model, (1) the cross section of the pulvinus is a perfect circle with a diameter *D*, (2) the cross sections of the upper (adaxial) and lower (abaxial) motor tissues are perfect circles with a diameter *D/2*, (3) the turgor pressure of the adaxial motor cells *P_ad_* and that of the abaxial motor cells *P_ab_* are different in deformed pulvini, and (4) *P_ab_* decreases in drooping leaves. Assuming that the pressure *P_ab_* decreases, it is concluded that *M* is proportional to both the difference between *P_ad_* and *P_ab_* (Δ*P*) and the cube of the diameter *D*.

(12) $$M=\beta \cdot \Delta P\cdot D^{3}$$

where

(13) $$\Delta P=P_{ad}-P_{ab}$$

and *β* is a constant that compensates for deviations caused by uncertainties in the area of motor cells and other factors. If *P_ad_* is not changed, a decrease in *P_ab_* causes an increase in Δ*P*, resulting in pulvinar deformation. In a previous study by Kagawa and Saito, a value for *M* for the leaf movement in *Mimosa pudica* was calculated based on a mechanical test, from which the authors estimated the value of ∆*p* to be 1.3 MPa [12]. This *∆p* value was close to that obtained in a previous study [13], suggesting that their model was valid. This study led to recent theoretical and biomimetic studies on the rapid leaf movement of *M. pudica* [14,15,16,17].

**Figure 4:**
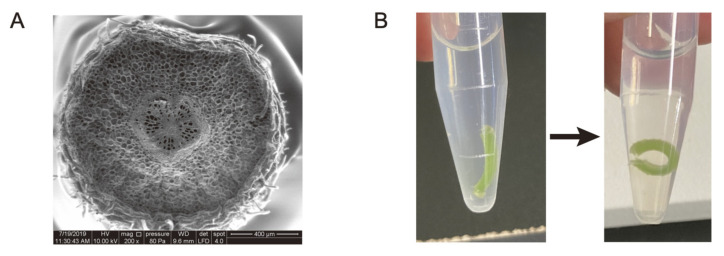
Cross section of a pulvinus observed by scanning electron microscopy (**A**) and bending deformation of tissue slices of motor cells immediately after (**B**, **left**) and after >20 min (**B**, **right**) of soaking in hypotonic solution. (**A**), *Desmodium paniculatum*; (**B**), *Pueraria montana var. lobata*.

In Kagawa and Saito’s model, the total turgor pressure generated by the motor cell populations in the adaxial or abaxial cross sections was applied to the center of each. In other words, the differences in the spatial contribution of the turgor pressure within each population were ignored. However, as described above, when bending deformation occurs due to an external force, the area near the center line does not expand or contract, and the degree of expansion or contraction increases as the distance from the center line increases. Furthermore, as the degree of expansion or contraction increases with distance from the center line, so does the conversion efficiency to bending deformation. Indeed, in cross sections of the pulvinus, it can be seen that the rigid vascular bundle is located almost in the center, hindering expansion and contraction near the center line (Figure 4A). In fact, real-time imaging of *M. pudica* leaves by X-ray-computed tomography showed almost no expansion or contraction of the xylem in the pulvinus during deformation [18]. Even if the motor cells near the vascular bundle could expand as much as those at the periphery of the pulvinus, such deformation would not only waste energy, but also risk shear failure because of the significantly lower extensibility of the vascular bundle relative to the neighboring motor cells. In preliminary experiments, a thick tangential tissue slice of motor cells immersed in a hypotonic solution underwent bending deformation (Figure 4B), implying that the mechanical properties of the motor cells at the center and the periphery of the pulvinus may differ. If this phenomenon is supported by further investigation in a variety of legume species, it is expected to be a new topic for future research into pulvinar deformation.

## 4. Molecular Genetic Studies of the Pulvinus

How are such unique physiological and mechanical features of pulvini established during leaf development? The LATERAL ORGAN BOUNDARIES DOMAIN (LBD) transcription factor (TF) ELONGATED PETIOLULE1 (ELP1)/PETIOLULE-LIKE PULVINUS (PLP) plays a central role in the establishment of pulvinar identity. ELP1/PLP has been identified as a master regulator of pulvinus development in the model legume *Medicago truncatula*. An *elp1*/*plp* mutant of *M. truncatula* lacks a motor organ and develops a petiolule-like organ instead [19,20]. The pulvinus of a wild-type plant has numerous small and compacted motor cells, clearly distinguishing the pulvinus from other axial organs. In the mutant, the corresponding tissue contains cells that are elongated along the proximodistal axis, as in other axial organs [19]. In addition, transgenic *M. truncatula* plants overexpressing *ELP1*/*PLP* had markedly shortened stems and petioles [19]. Taken together, these results clearly show that the pulvinus and the petiole/petiolule are homologous organs. They also suggest that the *ELP1*/*PLP* gene regulates cell elongation and, therefore, the development of the distinctive small and compacted motor cells. Furthermore, molecular genetic analysis of *sleepless* (*slp*) mutants in *Lotus japonicus* and *apulvinic* (*apu*) mutants in *Pisum sativum*, which show phenotypes similar to that of the *elp1*/*plp* mutant, revealed that *SLP* and *APU* are orthologs of *ELP1*/*PLP* [19]. This suggests that the regulation of pulvinus development by the master gene *ELP1*/*PLP* (or an ortholog) is a universal mechanism among legumes.

The study of *ELP1*/*PLP* also clarified the role of auxin in pulvinar development. In plants transformed with a DR5-promoter-driven GFP construct (an auxin reporter), GFP fluorescence was observed in the pulvinus in the wild type but not in the corresponding part of the *elp1* mutant [20,21]. Furthermore, the auxin biosynthetic gene *YUCCA10* and auxin-responsive genes in the F-box protein TIR1-dependent auxin signaling pathway, such as *ARF16*, *IAA14*, *SAUR14/48*, and *GH3.5*, were downregulated in the *elp1-1* mutant [21]. The treatment of *M. truncatula* with yucasin, a specific chemical inhibitor of the YUCCA protein, inhibited normal pulvinus development [21]. In another study, Zhou et al. found that a mutant of the F-box protein MIO1/SLB1 developed a decreased number of motor cells and exhibited defective leaf movement [22], suggesting that the size of the pulvinus was regulated by ubiquitin-mediated target degradation in addition to the TIR1 pathway. Taken together, it can be concluded that auxin is involved in the regulation of pulvinus development.

Brassinosteroids are also becoming known as regulators of pulvinar development downstream of the ELP1/PLP pathway. Kong et al. found that overexpression of ELP1/PLP activated the expression of BAS1, an inhibitor of brassinosteroid biosynthesis, indicating that the brassinosteroid signaling pathway played a role downstream of ELP1/PLP [23]. Through forward genetics, Zhao et al. obtained a nyctinastic movement-deficient mutant of *M. truncatula*, *mtdwf4* [24]. Kong et al. and Zhao et al. independently reported that mutants of *M. truncatula MtDWF4*, which is an ortholog of *Arabidopsis thaliana DWF4*, were defective in closing leaves at night [23,24]. In addition, Zhao et al. demonstrated that the nyctinastic movement of the leaflets was rescued by exogenous application of the brassinolide (BL) 24-epi-BL once a day for 2 weeks and the movement of the terminal leaflet was rescued by removing two lateral leaflets [24], indicating that the phenotype resulted from physical constraints caused by the deformation of leaves. Moreover, Kong et al. found that the application of propiconazole, which is an inhibitor of brassinosteroid biosynthesis, to wild-type plants mimicked the *p35S::ELP1/PLP* phenotype, including curled leaves, irregularly shaped pulvinar motor cells, a reduced length of the pulvinus, and defects in nyctinastic movements [23]. These results suggest that the morphology of the pulvinus is regulated by a brassinosteroid-dependent signaling pathway.

As mentioned above, changes in the size of the motor cells are caused by changes in ion concentration gradients mediated by ion and water channels, a process that has been studied intensively mainly using biochemical or electrophysiological approaches. The roles of ion channels in leaf movement are well summarized by Moran [25] and by Ueda et al. [26]. In *M. pudica*, the activity of mechanosensitive ion channels was detected in protoplasts isolated from these cells harvested from the pulvinar region of the leaflet and the activity was required for the mechanically evoked leaflet closure [27]. The light response regulating pulvinar movement was also briefly covered by Moran [25]. Electrical/calcium signaling in the regulation of leaf movement was reviewed by Moran [25], by Hagihara and Toyota [28], and by Mano and Hasebe [29]. Due to the technical difficulty of generating transgenic legumes, previous studies on leaf opening and closing in legumes have been dominated by indirect biochemical approaches, such as heterologous expression in Xenopus oocytes. However, Oikawa et al. successfully used transient virus-induced gene silencing to directly demonstrate that the anion channel protein SLAH contributed to leaf movement in *Glycine max* [30].

In addition to studies related to channel proteins, Kanzawa et al. showed that tyrosine residues in the actin of motor cells in the main pulvinus were related to the seismonastic movement (quick leaf folding in response to touch) of *M. pudica* [31]. The tyrosines were phosphorylated before the movement and dephosphorylated after the movement. They also showed that the actin filaments were fragmented after movement and that the inhibition of the dephosphorylation or fragmentation processes inhibited the rapid leaf movement [31]. How these actin modifications are involved in seismonastic leaf movements and whether actin also plays a role in nyctinastic movements is not yet clear and awaits further study.

Pulvini (i.e., joint-like thickenings) at the end(s) of petioles are not specific to legume species, but also found in nonlegume plants [32,33,34]. Pulvini in some nonlegume plants are closely related to leaf movement [32,33] as in legume species. Interestingly, the expression of a *Vitis vinifera* K^+^ channel VvK3.1 found at the abaxial parenchyma cells of pulvini suggests that the regulation of ion channels may be related to leaf movement even in nonlegume plants [32]. Meanwhile, in many species including legume species, pulvini appear to be associated with leaf abscission [34,35]. In *M. truncatula*, ELP1/PLP is required for abscission zone formation in the pulvinus and modulates genes involved in hormonal homeostasis, cell wall remodeling, and degradation during abscission process [35]. The diversity of development and function of pulvini is an intriguing question from an evolutionary point of view.

## 5. Adaxial–Abaxial Leaf Patterning and the Pulvinus

As described above, the difference in turgor pressure between motor cells of the upper (adaxial) side versus the lower (abaxial) side is the driving force of the bending deformation of a pulvinus. By analogy to muscles, the inside of a bent pulvinus in a leaf asleep at night is called the extensor, and the other is called the flexor. Which side (adaxial or abaxial) corresponds to the extensor or flexor depends on the plant species and the degree of the pulvinus (main/primary, secondary, or tertiary; Figure 1). Extensor and flexor motor cells differ in the timing of ion fluxes and their morphological/mechanical properties [4,26]. Presumably, then, the cell fate for each side is determined by different mechanisms. What processes determine the differences in the properties of the adaxial and abaxial motor cells of a pulvinus? This section introduces what has been clarified so far regarding adaxial–abaxial patterning in early leaf development.

The adaxial and abaxial domains of the leaf are set up early in the leaf development. The establishment of leaf polarity has been well studied in model plants such as *A. thaliana*, but only an outline of the findings is given here because several other reviews have covered this topic [36,37,38,39]. The leaves start to develop from primordia on the periphery of the shoot apical meristem (SAM), which includes a group of stem cells. The side of the leaf primordium closest to the SAM becomes the adaxial side of the leaf, and the side farthest from the SAM becomes the abaxial side. The developing leaf first elongates by apical growth, then the leaf blade is formed by marginal growth. As the leaf develops, the cells in its epidermis, mesophyll, and vascular bundle differentiate in distinct ways depending on their positions relative to the adaxial and abaxial sides of the leaf. For example, adaxial mesophyll cells elongate along the adaxial–abaxial axis and are densely packed, while the abaxial mesophyll cells develop a complex shape and the tissue has many air spaces. In the vascular bundle, the adaxial and abaxial cells differentiate into xylem and phloem cells, respectively. TFs specifically expressed on the adaxial or abaxial side of the developing leaf have a prominent role in establishing and maintaining the adaxial/abaxial pattern and adaxial/abaxial differentiation. Among these TFs, the adaxial regulators include ASYMMETRIC LEAVES2 (AS2) and Class III HD-ZIP (HD-ZIPIII) family TFs, and the abaxial regulators include KANADI family TFs, AUXIN RESPONSE REGULATOR3 and 4 (ARF3/4), and YABBY family TFs. In addition, the specific adaxial/abaxial expression and/or function of these TFs are directly or indirectly regulated by small RNAs, transcription cofactors, competitive factors, translation-related factors, and plant hormones. The middle domain of the leaf is established at the boundary between the adaxial and abaxial domains, and interactions among the TFs specific to these three domains are important for maintaining the adaxial/abaxial pattern. The expression of WUSCHEL-RELATED HOMEOBOX1 and 3 (WOX1/3) TFs is induced in the region where the abaxial-biased distribution of auxin overlaps with the adaxial function of MONOPTEROS/ARF5, resulting in the establishment of the middle domain [40]. WOX1/3 play an essential role in leaf flattening and cell fate determination in the leaf margin, as well as in the maintenance of the adaxial/abaxial pattern in cooperation with the auxin pathway [41,42]. It is becoming apparent that similar mechanisms function in early leaf development of the legume model plant *M. truncatula* [43,44,45].

Among these many adaxial/abaxial regulators, which ones are involved in the differentiation of each tissue? Do some TFs act alone to promote a cellular property of particular tissues, or do the TFs function cooperatively? Although the answers to these questions remain unknown, the phenotype of the *A. thaliana* double mutant *prs wox1* may be a clue. In this loss-of-function mutant of the middle domain regulators WOX1/3, some of the adaxial/abaxial-specific tissues near the leaf margin are mixed [46]. For example, the morphological features of the epidermis pavement cells normally found on the abaxial side extend across the leaf margin to the adaxial side. Conversely, the distribution of the trichomes, which is usually limited to the adaxial epidermis, extends to the abaxial side in the *prs wox1* mutant. This phenotype suggests that different adaxial/abaxial regulators independently affect the distribution of epidermal pavement cells and trichomes. In the *prs wox1* double mutant, the expression of *AS2* extends to the abaxial epidermis, while the region of HD-ZIPIII function is restricted to a narrower region of the adaxial domain due to the expansion of the promoter activity of *microRNA165a*, which binds to *HD-ZIPIII* transcripts and promotes their degradation [46,47]. These findings suggest that HD-ZIP III and AS2 are involved in different tissue differentiation pathways: HD-ZIP III is closely related to the cell fate of epidermal pavement cells, and AS2 is closely related to trichome distribution.

Let us return to the legume pulvinus. Cross sections of the pulvinus show that the dorsoventrality of the pulvinar tissues is very distinct from that of other parts of the leaf. First, although the adaxial surface is slightly flattened, the tissues of the pulvinus are arranged concentrically, forming an almost circular cross section (Figure 4A). The vascular bundles of the pulvinus are arranged with completely radial symmetry. These features are completely different from those of a petiole or leaf blade. In addition, the cortical motor cells and epidermis of the pulvinus are morphologically unique. Yet, despite the radial symmetry of the pulvinus, motor cells on the adaxial and abaxial sides are clearly different in terms of their mechanical properties and physiological response. What mechanism simultaneously establishes these contradictory characteristics, i.e., small morphological differences along with prominent mechanical and physiological differences? Answering this question will be an interesting topic for future research on leaf movement, especially from an evo–devo perspective.

## 6. Conclusions

Here, we briefly reviewed previous research on the reversible deformation of motor cells in legume pulvini. The reversible deformation of plant cells is an elastic process and can be driven by changes in turgor pressure resulting from differences between extracellular and intracellular osmotic pressure. The spring-like behavior of cellulose microfibrils in the pulvinar motor cells plays a central role in the proximodistal-biased deformation of the pulvinus, and the structure of the cell wall matrix is also important for enabling sufficient expansion/contraction within the physiological range of turgor pressure. The difference of turgor pressure between motor cells on the adaxial versus abaxial sides results in the bending moment of the pulvinus and dynamic movement of the attached leaf. The internal structure of the pulvinus, in particular the location of the vasculature, appears to be appropriate for an organ that undergoes bending deformation. Meanwhile, the mechanical properties of motor cells near the center of the pulvinus may differ from those of motor cells at the periphery, but this remains to be solved.

Recent genetic studies have shed light on the regulation of pulvinar development at a molecular level. ELP1/PLP is a master regulator of pulvinar development that confers the features of pulvinar tissues. Research on ELP1/PLP has also demonstrated that auxin and brassinosteroids have roles in pulvinar development downstream of ELP1/PLP. As we have seen so far, the development of the pulvinus is very similar to that of other plant organs such as leaves, stems, and roots in that their development is controlled by master genes. However, because the pulvinus is a motor organ, its morphology is continually and dynamically controlled by endogenous regulation and external stresses even after the completion of its development. Molecular genetic approaches are being used to investigate the regulation of molecules related to dynamic pulvinar deformation, e.g., ion channels and the actin cytoskeleton. The pulvinus has a unique dorsoventrality: motor cells of the adaxial and abaxial sides show differences in mechanical properties and physiological response, even though the pulvinus is radially symmetric from a morphological point of view.

As described above, the reversible deformation of pulvini and leaf movement are an interdisciplinary field that includes physics and chemistry as well as biology, so there may be challenges to overcome at the beginning of their study. However, this also means that this field is a treasure trove of unsolved mysteries. The mystery of plant movement has attracted attention ever since the era of Darwin. In the future, the combination of cutting-edge observation techniques, genetic manipulation, and informatics with traditional theory will answer the various questions mentioned in this review.

## Data Availability

Not applicable.
